# Aberration compensation in Doppler holography of the human eye fundus by subaperture signal correlation

**DOI:** 10.1364/BOE.528568

**Published:** 2024-09-04

**Authors:** Zofia Bratasz, Olivier Martinache, Julia Sverdlin, Damien Gatinel, Michael Atlan

**Affiliations:** 1 Institut Langevin, ESPCI Paris, PSL University, CNRS, 75005 Paris, France; 2 Quinze-Vingts National Eye Hospital, 28 rue de Charenton, Paris 75012, France; 3 Essilor Instruments, France; 4 Rothschild Ophthalmologic Foundation, Clinical Studies Department, Paris 75019, France

## Abstract

The process of obtaining images of capillary vessels in the human eye’s fundus using Doppler holography encounters difficulties due to ocular aberrations. To enhance the accuracy of these images, it is advantageous to apply an adaptive aberration correction technique. This study focuses on numerical Shack-Hartmann, which employs sub-pupil correlation as the wavefront sensing method. Application of this technique to Doppler holography encounters unique challenges due to the holographic detection properties. A detailed comparative analysis of the regularization technique against direct gradient integration in the estimation of aberrations is made. Two different reference images for the measurement of image shifts across subapertures are considered. The comparison reveals that direct gradient integration exhibits greater effectiveness in correcting asymmetrical aberrations.

## 1. Introduction

The prevalence of astigmatism, hyperopia and myopia among adults is estimated at 40.4%, 30.9% and 26.5%, respectively [1]. Hence, high-resolution imaging of the human eye fundus, particularly in clinical applications, requires a robust aberration correction technique. Many techniques currently employed benefit from regularization with a Zernike polynomials basis which accurately describes the aberration wavefront over the circular pupil. Moreover, the second radial order of Zernike polynomials statistically represents around 91% of the total root-mean-square wavefront error [2]. As a result, determining only 3 parameters considerably improves the resolution of the images obtained. Furthermore, Zernike polynomials up to 4th describe above 99% of of the total root-mean-square wavefront error [2].

Since wavefront distortion is a common problem in imaging, various compensation techniques have been developed to compensate for it. Several of them have been successfully adapted to ophthalmic devices. One of the most frequently used solutions is based on adaptive optics designed for stellar imaging [3–5]. Several reviews have treated the topic of adaptive optics applied to high resolution imaging [6–8]. Alternatively, the aberration compensation can be made by deconvolution of the total measured field [9,10]. However, for some ophthalmic applications, the guide star approach may be cumbersome. To overcome this issue, digital wavefront estimation in sub-pupils [11–14], and blind iterative phase compensation based on the quality of image features [15–17] were investigated for digital holographic imaging. The main limitation of wavefront sensing is the compromise between the area of wavefront sampled and the precision of slope estimation [17]. On the other hand a blind iterative approach has a longer computation time.

In this paper, it is demonstrated that digital wavefront estimation in subapertures [11], which is referred to as the numerical Shack-Hartmann method, can be applied to laser Doppler holography (LDH) [18] to improve resolution and reveal retinal capillaries. The numerical Shack-Hartman approach based on sub-pupil correlation is used to measure local slopes of the aberration wavefront. The estimation of the aberration wavefront from measured local gradients can be done by projection on a chosen finite basis. The authors of [11], in their initial paper, proposed using a Taylor monomials basis. However, for applications in the in-vivo imaging of the human retina with optical coherence tomography, a Zernike polynomial basis was chosen as a regularization technique [12,17]. Alternatively, the aberration wavefront can be estimated from the measured local slopes by direct gradient integration [14]. A comparative analysis between regularization via projection onto Zernike polynomials and direct gradient integration for aberration estimation is performed. The results obtained indicate that the latter is more robust in compensating asymmetrical aberrations in Doppler holography.

## 2. Experimental setup

**Fig. 1.**
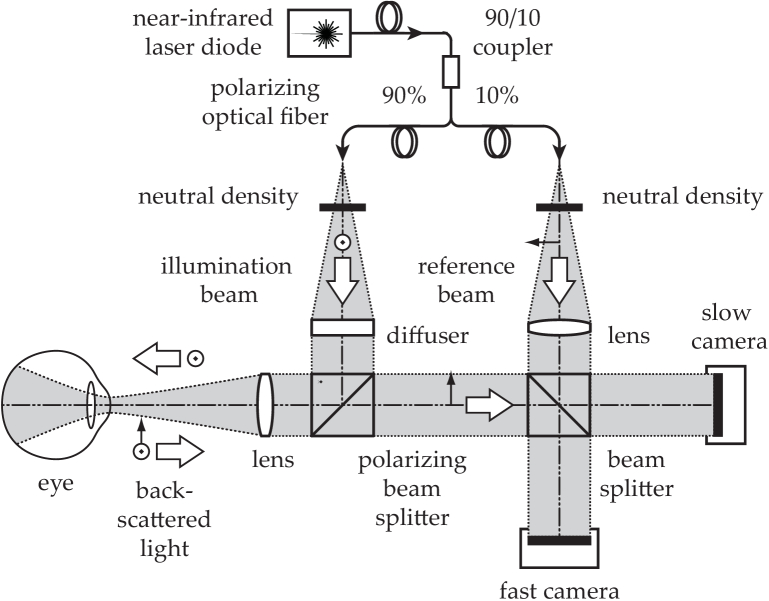
Sketch of the optical configuration. An inline Mach-Zenhder near-infrared laser interferometer mixes the light backscattered by the eye fundus with the reference beam. Two cameras record the optical interference patterns.

The optical configuration of the experimental device used for Doppler holography of the human retina is based on a Mach-Zehnder in-line interferometer [19] (Fig. 1). A laser beam is split into reference (10% of the optical power) and object arms (90% of the optical power). The latter passes through a diffuser (Thorlabs ED1-C20-MD, circle pattern engineered diffuser) and is focused by a lens (Thorlabs LB1630-B, focal length *f* = 100 mm) in front of the eye to maximise the area of the retina that is illuminated. The diffuser-to-eyepiece centre distance is ∼ 100 mm. The geometry of the system is based on two beam-splitting cubes, one of which is polarizing which enable the rejection of specular reflections. The reference beam passes through a lens (Thorlabs LB1676-B, focal length 100 mm) that shapes its wavefront in the detection (camera) planes to benefit from homogenous illumination across the sensor, and increase the blur rate of the twin holographic image in the numerical reconstruction. Two cameras are used to record the sequence of interferometric patterns: one serving for real-time preview (Ametek Phantom S710, frame rate: 5 kHz, pixel pitch: 20 *μ*m), the second for offline rendering (Ametek V2012, frame rate: 33 kHz, pixel pitch: 28 *μ*m). The sampling frequency is well above the established requirements for the phase stability in the human eye [20]. The most significant difference with previously reported configurations [18] is the presence of the optical diffuser that scatters the illumination beam.

**Fig. 2.**
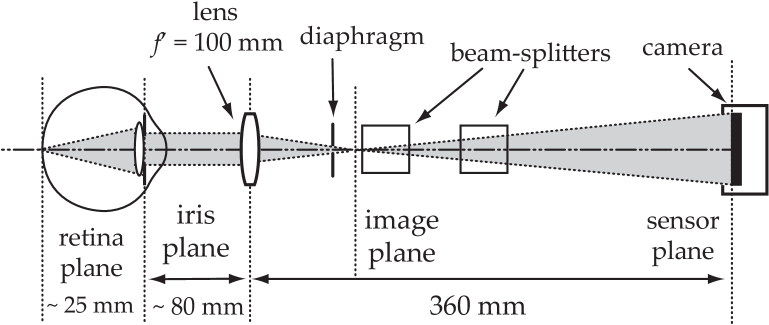
Scheme of optical configuration for holographic imaging of the eye fundus with diffuse laser illumination, including approximate optical conjugations. The field of view is given primarily by the magnification of the lens but can be affected by the image of the pupil acting like a field diaphragm.

The wavelength of the near infrared laser is λ=852nm , the pixel pitch of the offline camera used to sample all the frames used as raw data in this study is d=28μm , the average distance between the volunteer’s cornea and retina $d_{eye}=25\;mm$ , the number of lateral pixels of the sensor frame in each direction is $N_{x}=768$ . The subject’s pupil is located at a distance of approximately 80 mm from the eyepiece (Fig. 2). The image of the retina is formed with a $M=f/d_{eye}=100/25=4$ magnification ratio, at ≈ 260 mm from the camera. The pixel pitch of the numerically rendered image in the sensor plane via the angular spectrum propagation method for reconstruction distances $z<N_{x}d^{2}/\lambda ≃0.71\;m$ has a constant value of 28 *μm*. The resulting field of view in the retina plane is equal to $N_{x}d/M=5.375\;\text{mm}$ , which corresponds to the field of $\approx 12^{\circ }$ .

## 3. Digital image rendering

A 512-frame sequence of 12-bit, 768-by-768-pixel interferograms recorded at a rate of 33,000 frames per second, is numerically propagated using of the angular spectrum method [18] to reconstruct holograms of the eye fundus with a distance parameter z∼0.35 . After singular value decomposition filtering, applied to reject the principal components of largest intensity [21], the power Doppler signal in the band 3-16.5 kHz is calculated using short-time Fourier transformation with a cutoff frequency of 3 kHz. This standard image rendering procedure is performed with the open-source image rendering software http://www.holowaves.org/ (branch : master, commit : 17df875).

**Fig. 3.**
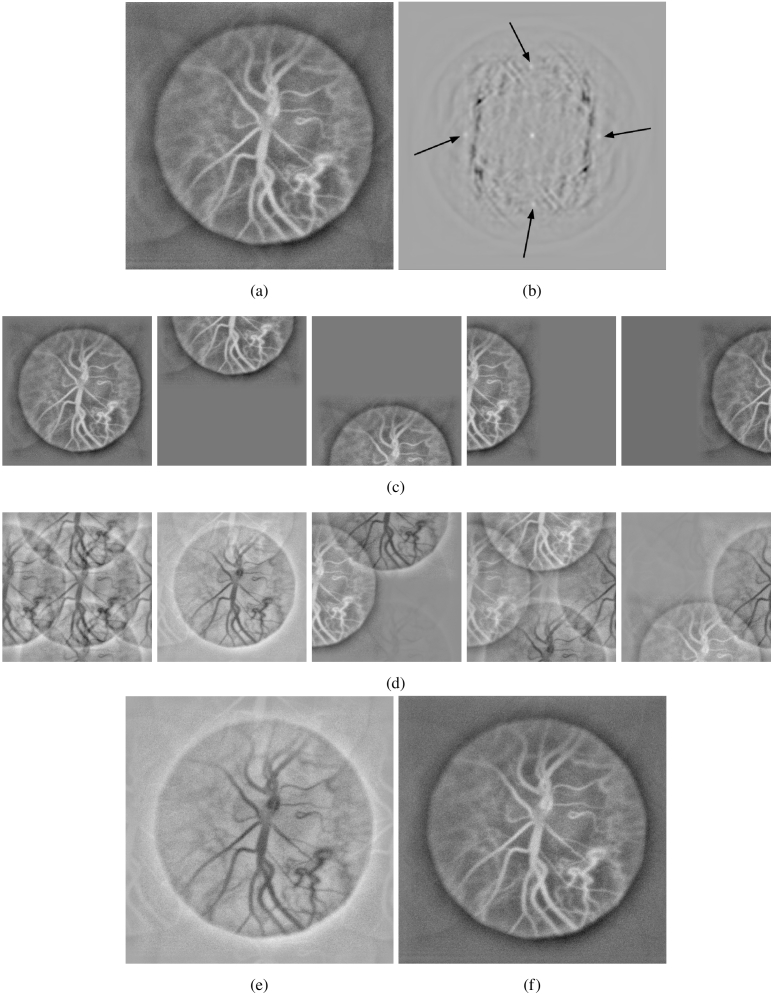
3a Image of the eye fundus with ghost images in four directions. 3b Cross-correlation function of the initial image allowing to find replicas positions. 3c Basis of images shifted to the position of the replicas. 3d SVD decomposition of the previous basis. 3e Replica image to be subtracted. 3f The same image with replicas attenuated using singular values decomposition approach.

Four image aliases may appear at horizontal and vertical offsets as seen in Fig. 3(a) due to the excessive difference between curvatures of the reference and object optical waves interfering at the sensor array. To filter out their presence in post-processing, the following procedure is carried on. Firstly, the position of replica is determined by the local maxima of the cross-correlation function of the rendered image (Fig. 3(b)). Next, a basis of images shifted to the positions of the replicas is created (Fig. 3(c)). A singular value decomposition of this basis creates a set of eigenvectors that carry the spurious image features to be removed (Fig. 3(b)). We subtract from the images with shifted features the first eigenvector of the SVD, and we sum them up to create the image to be removed (Fig. 3(e)) from the original. Finally, we obtain an image where replicas intensity is attenuated (Fig. 3(f)). The attenuation can be quantified by comparing the ratio of secondary peaks with respect to the maximal value of the cross-correlation function before and after correction. Using this method we can evaluate that the intensity of top and left replicas initially represented 7.85% of the main features and decreased 2.75 times. The bottom and right replicas had an initial intensity of 3.76% (with respect to the main features) which decreased 2.08 times. The code used to obtain those results can be found in the open-source repository https://github.com/DigitalHolography/ImageReplicaRemoval (branch : master, commit : 9481839).

## 4. Numerical estimation of the aberration via subaperture signal correlation

### 4.1. Method

The combination of the recording system and signal processing performed in LDH [18] enables the extraction of the complex object field information from optically-acquired on-axis interferograms. The algorithm of numerical phase correction for interferometric full-field imaging systems based on subaperture correlation [11] can hence be used in Doppler image rendering routines to perform aberration correction.

Let $A_{0}(x,y,z)$ and A(x,y,z) stand for the wavefront function in the Fourier plane before and after deformation. Using isoplanatic approximation, that wavefront can be expressed as:


(1) $$A(x,y)=A_{0}(x,y)e^{i\left(\Phi (x,y)-\Phi^{corr}(x,y)\right)}=A_{0}(x,y)e^{i\Phi^{res}(x,y)}$$

where Φ(x,y) is the initial phase in the pupil plane with aberrations, $\Phi^{corr}(x,y)$ is the wavefront corrector and $\Phi^{res}(x,y)$ is referred to as the residual phase. The error of the aberration estimation can be assessed by calculation of the variance of residual phase. The subaperture images correlation algorithm [11] finds the $\Phi^{corr}(x,y)$ function in the pupil plane, by approximating the wavefront with a finite number of inclined plates (Shack-Hartmann wavefront sensing method). Additional regularization can be performed assuming that the aberration phase can be represented as a linear combination of a series of known functions, for instance:


(2) $$\Phi^{corr}(x,y)=\sum_{j}c_{j}Z_{j}(x,y)$$

where $Z_{j}$ are Zernike polynomials.

### 4.2. Implementation

**Fig. 4.**
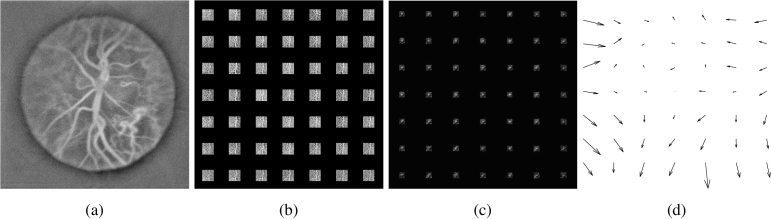
Diagram illustrating steps of the numerical Shack-Hartmann algorithm. 4a Image of the retina rendered without aberration correction. 4b Images rendered from fields in subapertures with masked outer parts. 4c Cross-correlations between subimages. 4d Shift vectors of the subimages.

The digital optical field in the image plane is obtained by angular spectrum propagation from each recorded interferogram (Fig. 4(a)). This field is then propagated to the reciprocal plane of the reconstructed image by spatial Fourier transformation and divided into regularly-spaced square apertures. Within each subaperture, the local wavefront distortion is approximated by a plane, tilted phase. The signal from each subaperture is processed separately to obtain an image by spatial Fourier transformation, singular value decomposition filtering [21], and short-time Fourier transformation over the 512-frame sequence to reveal a power Doppler image in the band 3-16.5 kHz (Fig. 4(b)), whose field of view corresponds to that of the initial image but its resolution is decreased due to the support reduction in the aperture plane. In the first approximation, the aberration wavefront modifies images by shifting them respectively according to local phase tilts. Those translations are quantified using the cross-correlation function (Fig. 4(c)) between reconstructed images from subapertures. A subpixel precision of shift estimation is achieved by fitting a parabola to the peak found. Additionally, when working with data with low signal-to-noise ratio, it can be advantageous to reject certain subapertures over which the cross-correlation peak value remains under the chosen threshold, meaning that resemblance between the features is low. In the end, the aberration wavefront is assessed from the measured vector of shifts from each subaperture (Eq. (4)), where *x* and *y* dimensions are processed independently.

To express the aberration wavefront as a linear combination of *p* first Zernike polynomials, a matrix of transition between two bases is calculated. Each Zernike polynomial is split into subapertures, over which the local gradient is calculated. Obtained shifts in the subapertures are assembled to form a n×p matrix M (Eq. (3)) representing *p* Zernike polynomials in the base of *n* couples of horizontal and vertical shifts between the subimages. Due to the circular vignetting of Zernike basis functions, subapertures from the corners are removed from the list of *n* measured shifts. The criterion of exclusion is based on assessment of the subaperture centre position with respect to the circle.


(3) $$M=\left(\begin{array}{cccc} m_{11} & m_{12} & \cdots & m_{1p}\\ m_{21} & m_{22} & \cdots & m_{2p}\\ \vdots & \vdots & \ddots & \vdots \\ m_{n1} & m_{n2} & \cdots & m_{np} \end{array}\right)$$

(4) $$y=\left(\begin{array}{c} y_{1}\\ y_{2}\\ \vdots \\ y_{n} \end{array}\right)$$

The Zernike coefficients $c_{j}$ can be found by solving (Eq. (5)).


(5) $$c={\left(M\right)}^{-1}y$$

After having estimated coefficients, the image in the reciprocal plane is multiplied by $e^{-i\Phi }$ phase to correct the aberrations.

The second regularization used in this paper is based on direct wavefront reconstruction from the gradient, without solving the linear equation. The respective shifts are first normalized (divided by the number of pixels in the subaperture) and then multiplied by πNx , where Nx is the size in pixels of the wavefront we wish to reconstruct, interpolated in both directions to acquire smooth function and integrated numerically [22].

### 4.3. Choice of the reference image

**Fig. 5.**
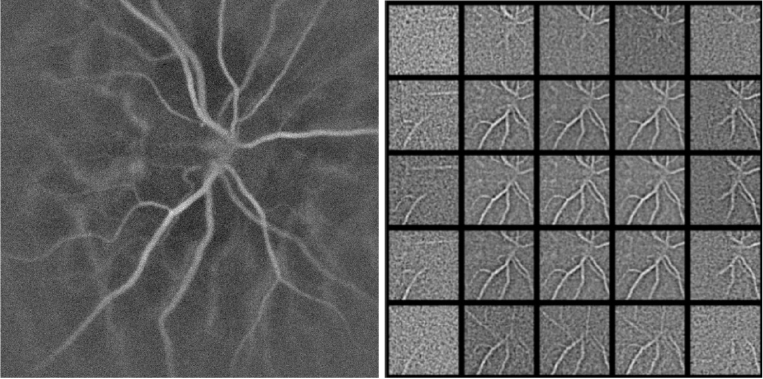
Images of a optic never head of a volunteer calculated from the entire pupil (left) and from subapertures in the pupil (right), where the position of the image corresponds to the position and size of the subaperture in the pupil plane.

The shifts are measured from the cross-correlation between two images reconstructed from subapertures. As a result, shifts are known up to a constant, which depends on the shift of the reference image. Since the linear terms in the wavefront function do not blur the image, the choice of the constant does not compromise the resolution of the output. The use of the central subaperture as the reference image [11] is advantageous because it usually holds the best-resolved image. Alternatively, [17] proposed cross-correlating randomly chosen subapertures which increased both the stability and the performance of the aberration measurement. However this approach cannot be easily applied to LDH as the angular composition is not homogeneous across the field of view (Fig. 5). As a result, two randomly chosen subapertures do not necessarily have enough features in common to allow for the unequivocal shift assessment. To avoid the cross-correlation being driven by the numerical artefacts that appear due to this effect, the subimages are cropped to the central part. The margin applied is chosen empirically and depends on the propagation distance. Typically the margin is adapted to make sure that the extension of features visible is similar for all subimages.

Alternatively, we propose the use of the original image resized to the dimensions of the subapertures as the reference image. The respective shifts can then be assessed for all subimages including the central one. This choice is motivated by a strong speckle pattern that appears in subapertures due to the fully coherent illumination. As this speckle pattern changes from one subimage to another, it can jeopardize the measurement of the respective shifts. Furthermore, choosing the resized image as the reference can be seen as setting the average gradient as a reference zero level, which ensures that there is no tip or tilt component in the aberration wavefront. Such a component would have no impact on the results if the regularization basis were perfectly orthogonal, but discretization through the system of subapertures introduces a spurious cross-talk between the modes.

## 5. Results

### 5.1. Measurement of a numerically imposed aberration using Zernike regularization method

**Fig. 6.**
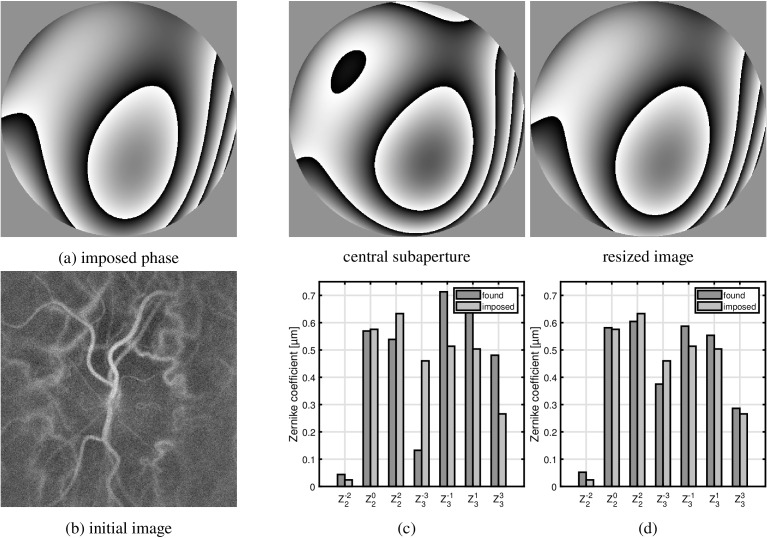
Wavefront distortion imposed (Fig. 6(a)) and found using subaperure correlation based algorithm with central subaperture (Fig. 6(c)) and resized image (Fig. 6(d)) used as the reference. Below, associated Zernike coefficients are plotted.

The LDH is used to image the optic nerve head of a volunteer. Experimental procedures adhered to the tenets of the Declaration of Helsinki, and the study was approved by an ethical committee (Comité de Protection des Personnes; clinical trial NCT04129021). Written informed consent was obtained from the subject. During reconstruction, the field in the Fourier plane is multiplied by a known phase function, which represents a linear combination of rank 2nd and 3rd of Zernike polynomials with randomly chosen coefficients. Consequently the wavefront distortion is measured using the algorithm described in section 4. Employment of both reference images is considered. The shifts are measured for 7 x 7 subapertures, and the wavefront estimated is projected onto Zernike polynomials from 2nd to 3rd degree. In Fig. 6 we compare the ground truth with the results obtained. To quantify the error of the wavefront estimation we calculate the RMS of Zernike coefficients for the imposed and residual phases. Using the central subaperture as the reference image enables decreasing of initial RMS of 1.73 *μ*m to 0.44 *μ*m (which corresponds to 26% of the initial value), whereas with the resized image used as a reference one obtains the final phase of RMS 0.25 *μ*m (15% of the initial phase).

### 5.2. Typical astigmatism correction for laser Doppler images with Zernike regularization approach

**Fig. 7.**
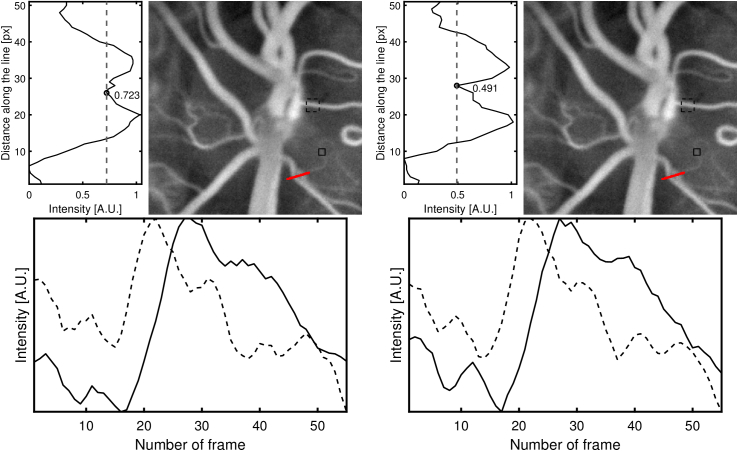
Images of a optic nerve head of a volunteer taken with a cylindrical lens of 1.25 D power placed in front of the eye, without (left) and with (right) numerical Shack-Hartmann aberration correction applied. On the left side of the images, the intensity profiles along the red lines are represented. To quantify the contrast, the ratio between the local minimum and the averaged value of the two peaks is calculated. Plots below the images represent the average temporal signals corresponding to the regions marked with dashed and continuous lines respectively.

The impact of aberration compensation on the images reconstructed is investigated. Wavefront distortion is induced by placing a lens with 1.25 D astigmatism in from of volunteer’s eye, which mimics the case in which aberrations are not circular functions perfectly inscribed in the field of view in the pupil plane. No additional aberration is added numerically. As previously the wavefront distortion is estimated in the reconstruction process using the numerical Shack-Hartmann algorithm, with the resized image used as a reference image. The field in the Fourier plane is split into 7 x 7 subapertures, and the wavefront estimate is projected onto Zernike polynomials from 2nd to 3rd degree. To compare the resolutions obtained for videos rendered with and without employing aberration correction algorithm, all frames calculated are averaged and a profile of the intensity along the line running through a region where two vessels run very closely to one another is plotted (Fig. 7). In addition, the influence of the resolution on the temporal signal is evaluated by comparison between two vessels of different sizes. The aberration correction technique allowed to considerably increase the contrast of the image calculated and, as a result, it facilitated the distinction of capillary vessels. There is no striking difference between the pulses detected.

### 5.3. Comparison of aberration correction robustness for different reference images and regularization techniques

#### 5.3.1. Numerically imposed aberration

**Fig. 8.**
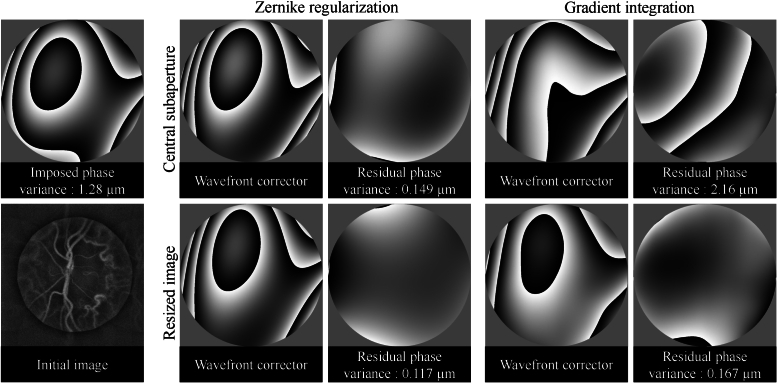
On a batch of interferograms allowing to obtain an image of the ONH of a volunteer (initial image) a numerical aberration is applied (imposed phase). Shifts between subimages are assessed with central subaperture or resized image used as a reference. Zernike regularization and gradient integration techniques are compared. For four combination possible we represent both wavefront corrector and residual phase with its variance.

Gradient integration and Zernike regularization methods are employed to reconstruct aberration numerically imposed on a batch of interferograms recorded with LDH and measured with the digital Shack-Hartmann algorithm. The distortion phase is constructed from Zernike polynomials of 2nd and 3th rank with coefficients chosen randomly between -1 and 1 *μ*m. For both techniques two possible reference images - central subaperture and resized full-pupil image - were tested during the assessments of shifts in subapertures. The comparison is done through estimation of the residual phase and its variance. Prior to these calculations, tilt and tip components - which have no influence on the resolution of the image obtained but can appear with the gradient integration approach - were subtracted. Immediate observation can be made that the gradient integration method with central subaperture used as a reference fails to correctly assess the aberration phase, however it manages to do so with resized image used as a reference Fig. 8. The Zernike regularization performs considerably better than the gradient integration approach. The observation from the Section 5.1 suggesting that the use of resized image as a reference improves the results has been confirmed. In this case the variance of the residual phase was 21% lower than that obtained using the central subaperture.

#### 5.3.2. Physically imposed aberration

**Fig. 9.**
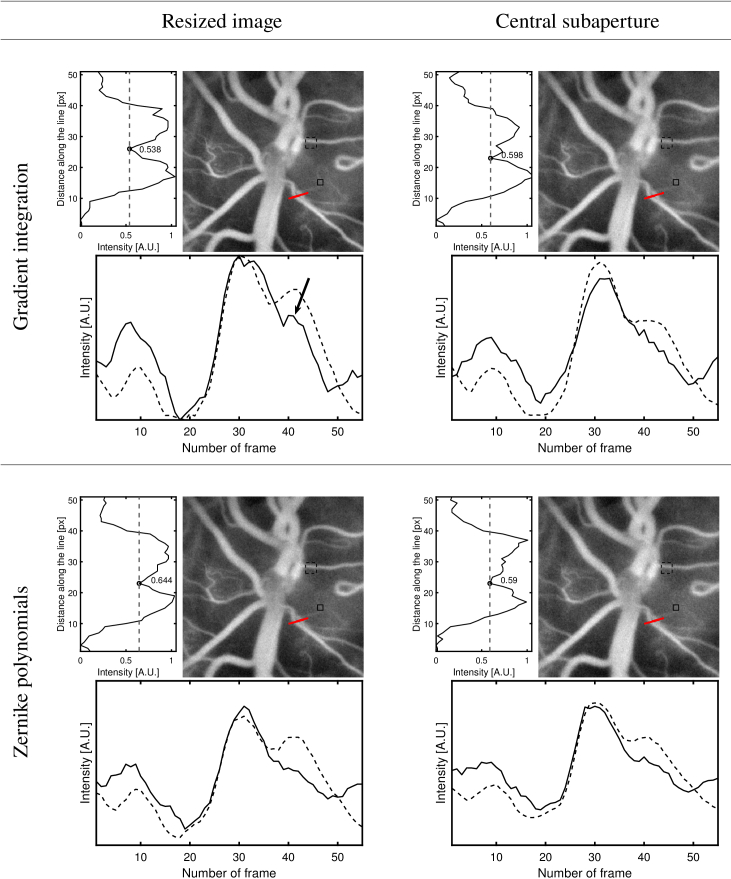
A comparison of close-up LDH images of a ONH of a volunteer reconstructed with numerical Shack-Hartmann aberration correction, for various regularization and shift assessment techniques. The profiles on the left side of the images correspond to the intensity profiles along the red lines indicated in the pictures. Plots below the images represent the average temporal signals corresponding to the regions marked with dashed and continuous lines respectively. An arrow in the top left pulse plot points to the dicrotic notch visible in the micro-vasculature pulsation waveform.

In the subsequent experiment a 6 D astigmatism lens was placed in front of the volunteer’s eye. The field in the Fourier plane is split into 10 x 10 subapertures, and the wavefront estimate is projected onto Zernike polynomials from 2nd to 5th degree. In total 18 coefficients are determined. The comparison between employing a central subaperture and the entire image resized as a reference image in the context of both regularization methods is illustrated in Fig. 9. For both regularization techniques, the use of resized image as a reference image helped reveal capillaries. The same criteria as in the section above are used to evaluate the resolution obtained for both the averaged image and the temporal signal measured. The two vessels are most easily distinguished for direct gradient integration wavefront reconstruction, with resized image used as a reference. For the Zernike regularization technique, the use of central subaperture as reference resulted in slightly better contrast, however the velocity profiles across the vessels seems overly noisy. For the temporal signals we can see that, once again, the direct gradient integration wavefront reconstruction, with resized image used as a reference, provided the best results. It is the only pulse profile for which the dicrotic notch can be detected for the smaller vessel.

#### 5.3.3. Physically imposed aberration with low stability

**Fig. 10.**
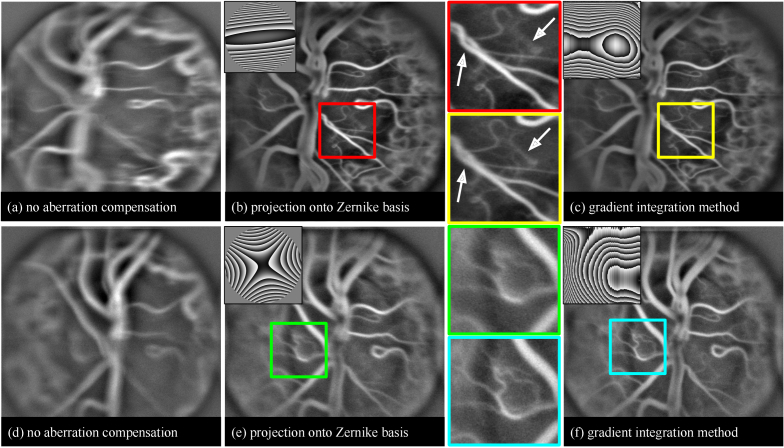
Doppler holographic images of the papillary region of the retina in a volunteer who holds an astigmatism 6D lens in front of his/her eye. The experiment is repeated twice (a, d). During image reconstruction we apply numerical Shack-Hartmann algorithm to measure aberration wavefront. In images (b, e) wavefront is projected on the Zernike polynomial basis, in (c, f) it is reconstructed using gradient integration technique. Images represented above are averaged over multiple frames of a video.

So far, the configurations with perfect alignment between the abberating lens and the optical paths have been considered. However, this model is frequently inadequate when imaging the human eye, as the patient moves and aberrations induced are not perfectly radially symmetrical. To mimic the above problems, a volunteer is asked to hold a 6D astigmatism lens in front of his/her eye. The wavefront is reconstructed, like previously, using the subaperture correlation technique. The shifts were estimated over 7 x 7 subapertures, using resized image as reference. The effectiveness of wavefront reconstruction through projection on the Zernike basis of 2nd order and direct gradient integration are compared. Two acquisitions are considered, images integrated over the total number of frames available are illustrated in Fig. 10. First video consists of 85 and the second of 100 frames. The duration of the measurement was determined by the time over which the subject was capable of focusing vision on one point. As one can see, imposing 6D astigmatism blurs the image of the optic nerve head reconstructed at an adapted focal depth (Fig. 10(a),(d)) and hinders the visualisation of capillary vessels. Generally, aberration compensation with regularization through the Zernike polynomials increases the resolution of the image, but its effectiveness varies between measurements (Fig. 10(b),(e)). Gradient integration technique, on the other hand, allows to repeatedly obtain images with high resolution (Fig. 10(c),(f)). The main difference between these two sets of data lies in the symmetry of the wavefront to be detected as shown in the inserts in the figure. In the first case (top), the aberrations are equally well approximated by symmetrical Zernike polynomials and direct gradient integration. The difference between the two results (Fig. 10(b),(c)) lies in various resolutions for superficial and deeper vessels. In the second case (bottom), the wavefronts reconstructed by the two techniques differ clearly.

## 6. Discussion and conclusions

This paper studies the deployment of aberration correction from subaperture image analysis for the LDH imaging of the human eye fundus. This technique consists principally of measuring the distorted wavefront in a discrete basis and regularizing it, in such a way that it represents a continuous function. Each of these steps can be adequately optimized and adapted to the imaging technique applied, which was the aim of this study.

One of the primary challenges met in deploying wavefront measurement from subaperture image analysis in digital holography is the varying angular composition of points across the field of view and the speckle pattern that emerges in images reconstructed from subapertures due to highly coherent illumination. To address this issue, we proposed to crop the subimages used for calculating cross-correlation function and use a resized full-pupil image as a reference for shift assessment. This approach favours accurate measurements of subimage shifts, a crucial first step in applying subaperture-based digital adaptive optics to laser Doppler imaging techniques. The study also compared regularization with Zernike polynomials and direct gradient integration approaches for the continuous wavefront reconstruction.

To begin with, the digital Shack-Hartmann algorithm was validated as a method for wavefront measurement for the LDH, as it correctly measured numerically imposed aberrations. Subsequently, it was observed that typical astigmatism (1.5 D) correction using the numerical Shack-Hartmann method with the Zernike regularization approach improved the resolution of the images obtained, but it has no significant impact on the temporal signal measured. The comparison of performance for the regularization and the gradient integration approach was conducted in three steps. Reconstructions of numerically imposed aberrations suggested that the Zernike regularization method performs far better than the gradient integration technique. For both wavefront reconstruction methods, resized images used as a reference performed better than the central subaperture. In the second step, videos were distorted with a 6 D astigmatism lens placed in front of the volunteer’s eye. The comparison between four possible combinations revealed that only the gradient integration technique with a resized image used as a reference allows the detection of the dicrotic notch in the microvasculature pulsation, which was visible in less distorted images. The difference between those results and the numerical experiment is probably caused by the fact that digitally imposed aberrations, constructed from the exact Zernike functions on which the wavefront is projected, create a bias towards the regularization approach.

Finally we studied a system where imposed aberrations are non-symmetrical and unstable in time. This allowed to mimic a configuration in which the optical axis of the eye and the setup are not aligned and subject struggles with fixation. In such a case, the wavefront reconstruction through gradient integration can perform considerably better, due to the higher degree of freedom and a higher stability of the measurement. In the Zernike regularization approach, the precision of shift measurements over each subaperture is critical; a single inaccurate measurement can compromise the entire wavefront reconstruction. Conversely, with gradient integration, an erroneous shift assessment in one subaperture remains a localised issue, thus preserving the overall accuracy of the wavefront reconstruction and minimising impact on image sharpness. It is possible that the effect of aberration correction is additionally enhanced by the registration algorithm, which performs better on sharper images.

In conclusion, we have seen that subaperture correlation based digital adaptive optics can be adapted to LDH allowing for better contrast and resolution in images obtained.Furthermore, we propose for the first time the use of resized full-pupil image as a reference instead of using one or multiple subapertures, which limits the error of the aberration estimation. In this study, we also compared two wavefront reconstruction methods, namely Zernike regularization and gradient integration, showing that the latter performs better in terms of resolution obtained for the case of LDH.

## Data Availability

Online repository for image rendering and aberration compensation routines [23]. Data used for this paper can be shared upon request.
